# Effects of recombinant human erythropoietin on cognition and neural activity in remitted patients with mood disorders and first-degree relatives of patients with psychiatric disorders: a study protocol for a randomized controlled trial

**DOI:** 10.1186/s13063-018-2995-7

**Published:** 2018-11-06

**Authors:** Jeff Zarp Petersen, Lejla Sjanic Schmidt, Maj Vinberg, Martin Balslev Jørgensen, Ida Hageman, Hannelore Ehrenreich, Gitte Moos Knudsen, Lars Vedel Kessing, Kamilla Woznica Miskowiak

**Affiliations:** 1 Neurocognition and Emotion in Affective Disorder (NEAD) Group, Copenhagen Affective Disorder Research Centre (CADIC), Psychiatric Centre Copenhagen, Copenhagen University Hospital, Blegdamsvej 9, DK-2100 Copenhagen, Denmark; 2 Department of Psychology, University of Copenhagen, Øster Farimagsgade 2A, DK-1353 Copenhagen, Denmark; 3 Clinical Neuroscience, Max Planck Institute of Experimental Medicine, Göttingen, Germany; 4 DFG Center for Nanoscale Microscopy & Molecular Physiology of the Brain (CNMPB), Göttingen, Germany; 5 Neurobiology Research Unit and Center for Integrated Molecular Imaging, Rigshospitalet, Copenhagen, Denmark; 6 Faculty of Health and Medical Sciences, University of Copenhagen, Copenhagen, Denmark

**Keywords:** Bipolar disorder, Depression, Cognition, Cognitive dysfunction, Erythropoietin, Pro-cognitive efficacy, Prefrontal cortex, Functional magnetic resonance imaging, Biomarker

## Abstract

**Background:**

Bipolar disorder (BD) and unipolar disorder (UD) are associated with cognitive deficits and abnormal neural activity in a “cognitive control network.” There is an increased prevalence of cognitive dysfunction in psychiatric patients’ first-degree relatives, which constitutes a risk factor for psychiatric illness onset. However, there is no treatment with enduring pro-cognitive efficacy. We found preliminary evidence for beneficial effects of eight weekly doses of recombinant human erythropoietin (EPO) on cognition in BD in a recent randomized controlled trial (RCT). The present RCT consists of two sub-studies that extend our previous work by investigating important novel aspects: (1) the effects of 12 weekly doses of EPO on cognition in first-degree relatives of patients with BD, UD, or schizophrenia; and (2) the effects of extending the treatment schedule from 8 to 12 weeks in remitted patients with BD or UD; and (3) assessment of early treatment-associated neural activity changes that may predict cognitive improvement.

**Methods:**

The trial comprises two parallel sub-studies with randomized, controlled, double-blinded, parallel group designs. First-degree relatives (sub-study 1; *n* = 52) and partially or fully remitted patients with BD or UD (sub-study 2; *n* = 52) with objectively verified cognitive dysfunction are randomized to receive weekly high-dose EPO (40,000 IU/mL) or placebo (saline) infusions for 12 weeks. Assessments of cognition and mood are conducted at baseline, after two weeks of treatment, after treatment completion, and at six-month follow-up. Functional magnetic resonance imaging (fMRI) is conducted at baseline and after two weeks of treatment. Psychosocial function is assessed at baseline, after treatment completion and six-month follow-up. The primary outcome is change in a cognitive composite score of attention, verbal memory, and executive functions. Statistical power of ≥ 80% is reached to detect a clinically relevant between-group difference by including 52 first-degree relatives and 52 patients with BD or UD, respectively. Behavioral data are analyzed with an intention-to-treat approach using mixed models. fMRI data are analyzed with the FMRIB Software Library.

**Discussion:**

If this trial reveals pro-cognitive effects of EPO, this may influence future treatment of mood disorders and/or preventive strategies in at-risk populations. The fMRI analyses may unravel key neurobiological targets for pro-cognitive treatment.

**Trial registration:**

ClinicalTrials.gov, NCT03315897. Registered on 20 October 2017.

**Electronic supplementary material:**

The online version of this article (10.1186/s13063-018-2995-7) contains supplementary material, which is available to authorized users.

## Background

Cognitive deficits occur in moderate to severe degree in patients with bipolar disorder (BD) and unipolar disorder (UD) [1–6]. These deficits are not only present during acute episodes, but commonly persist after remission [1–7]. This negatively affects patients’ quality of life, recovery rates, and socio-occupational functioning [6, 8–12] of which reduced work capacity is the largest area of socioeconomic burden [13, 14]. A higher occurrence of mild to moderate deficits has also been detected in BD, UD, and schizophrenia patients’ unaffected first-degree relatives compared with healthy controls with no first-degree family history of psychiatric disorder [15–19]. These impairments increase the risk of psychiatric illness onset in these individuals [20]. Cognition is therefore a key treatment priority in patients with mood disorders and genetically predisposed individuals [21]. Nevertheless, there are no existing treatments with solid and enduring efficacy on cognitive dysfunction in these populations. New candidate treatments have only produced disappointing or preliminary results [22]. This is partially related to major methodological challenges, including the absence of a sensitive brain-based biomarker model to detect the efficacy of candidate treatments in phase 1 and 2 clinical trials [23]. In fact, candidate drug treatment screening typically relies on animal models with compounds being directly moved into large-scale, costly clinical efficacy trials, in cases of beneficial effects in the animal model. However, detection of pro-cognitive efficacy in animal models has poor predictive value in clinical trials [24], which underlines the need for a more valid, sensitive biomarker model for pro-cognitive efficacy. Emerging evidence highlights blood-oxygen-level dependent (BOLD) functional magnetic resonance imaging (fMRI) response in the prefrontal cortex (PFC) as a promising biomarker for cognitive dysfunction and early cognitive improvement. In particular, aberrant (primarily hypo-) activity in the dorsolateral and medial PFC (dlPFC and mPFC) during working memory and episodic encoding tasks is the most reproducible neural marker of cognitive dysfunction across neuropsychiatric disorders, including BD, UD, schizophrenia, and genetically predisposed individuals [25–31]. This aberrant activity reflects difficulties with selecting and maintaining stimuli in working memory and strategic memory encoding [32]. Reversal of aberrant dorsal prefrontal activity may therefore constitute a promising neural biomarker for cognitive improvement.

Preclinical and clinical studies point to erythropoietin (EPO) as one of the most promising candidate cognitive enhancement treatments [33]. EPO is produced in the brain where it exerts neurotrophic and neuroprotective actions and plays a central role in cognitive functioning [33–37]. Clinical studies have found that repeated systematically administered high-dose recombinant human EPO versus placebo (saline) improves attention, memory, and executive functions after 8–12 weeks of treatment across neuropsychiatric disorders, including multiple sclerosis, Parkinson’s disease, schizophrenia, treatment-resistant depression (TRD; defined as failure to respond to ≥ 2 different types of antidepressant treatments given in sufficient doses over sufficient time [38]), and BD [39–43]. In particular, two randomized placebo-controlled trials from our group revealed that eight weekly EPO infusions improved several cognitive domains in patients with BD and TRD [39, 40]. This was accompanied by increased activity in dlPFC and dorsomedial PFC (dmPFC) during working memory and episodic encoding tasks [44, 45]. Notably, a single dose of EPO enhances cognition-related dlPFC and dmPFC activity without producing any change in red blood cells [46, 47]. This suggests that the EPO-associated increase in task-related dorsal PFC (dPFC) reflects direct effects of EPO in the brain. Taken together, these preliminary findings point to EPO as a promising cognition treatment and to neuronal activity change in the dPFC as a key neural correlate of treatment-related improvement of cognition. Nevertheless, our EPO trial had a number of methodological limitations [39, 40]. First, cognitive dysfunction was not verified with an objective (i.e. neuropsychological) measure before enrollment of participants. This is problematic since we found in post-hoc analyses that objective cognitive impairment at baseline was the strongest predictor of treatment success on cognition [48, 49]. Second, the primary outcome was a single measure of verbal memory, which contrasts with the recently published recommendations by the International Society for Bipolar Disorders (ISBD) Targeting Cognition Task Force that the primary outcome in cognition trials should be a broad cognitive composite score spanning attention, memory, and executive function [50]. Third, the lack of long-term follow-up assessment hampered insight into whether treatment-associated cognitive improvement was long-lasting. Fourth, we had not assessed functional capacity in the patient sample and thus had no insight into whether the EPO-related improvement of cognition translated into better functional capacity.

### Aims and hypotheses

The aims of the present EPO trial are threefold. First, we aim to investigate whether 12 weekly recombinant human EPO infusions ameliorate cognitive impairments in first-degree relatives without psychotic or mood disorders to patients with BD, recurrent UD, or schizophrenia (sub-study 1). This will allow for the first time to judge the disease-independent response of inherent cognitive genetic traits. Second, we will examine whether a longer treatment period is associated with similar or stronger cognitive improvement in remitted patients with BD or recurrent UD (defined as ≥ 2 treatment-requiring depressive episodes) (sub-study 2). Third, we will explore with fMRI the early neuronal changes that are predictive of subsequent clinically relevant cognitive improvement across these groups. We hypothesize that:

1. 12 weekly EPO infusions improve cognition in first-degree relatives and remitted patients with BD or recurrent UD in comparison with saline.
2. EPO versus saline-treated participants will display early cognition-related neural activity in dorsal PFC in the direction of the activity in healthy controls, which will correlate with cognitive improvement.

## Methods and design

### Participants

We will recruit 52–58 first-degree relatives without psychotic or mood disorders to patients with BD, recurrent UD, or schizophrenia (sub-study 1) and 52–58 patients with BD or recurrent UD in partial or full remission (defined as a score of ≤ 14 on the Hamilton Depression Rating Scale 17-items (HDRS-17; [51]) and the Young Mania Rating Scale (YMRS; [52]) (sub-study 2) with objectively verified cognitive dysfunction to obtain a complete dataset for 52 participants per sub-study (assuming a 10% drop-out rate from inclusion to treatment completion). Participants will be recruited from psychiatric centers in The Mental Health Services in the Capital Region of Denmark, consultant psychiatrists in the Capital Region of Denmark, as well as through advertisements on relevant websites. Within each sub-study, half of the participants will be randomized to receive active treatment (*n* = 26–29), while the other half will receive placebo (*n* = 26–29).

Eligible participants are aged 18–65 years, have fluent Danish skills, and display objectively verified cognitive dysfunction according to the Screen for Cognitive Impairment in Psychiatry (SCIP) [53–55]. Specifically, participants must have a total SCIP score of ≤ 77, which provides adequate sensitivity and specificity for cognitive impairment (86% and 70%, respectively) [53], or have a score corresponding to ≥ 1 standard deviation (SD) below the norm on ≥ 2 SCIP subtests [50, 53]. For individuals with a verbal IQ ≥ 120 (i.e. ≥ 1 SD higher than the average IQ in age-matched healthy control participants) according to the Danish Adult Reading Test (DART [56]), the adjusted inclusion criterion is a score > 1 SD below the norm on ≥ 1 SCIP subtest. In this way, cognitive impairment is to some degree established with reference to participants’ premorbid IQ in accordance with recent guidelines by the ISBD Targeting Cognition Task Force [50].

Patients are eligible if an ICD-10 diagnosis of BD or recurrent UD is confirmed with the Schedules for Clinical Assessment in Neuropsychiatry (SCAN) [57]. The maximum daily use of benzodiazepines allowed is 22.5 mg oxazepam (benzodiazepines will be avoided on the day of neuropsychological assessments). Patients’ medication must remain unchanged during the study period, unless a change of medication is deemed necessary by their treating psychiatrist. First-degree relatives are allowed to have minor psychiatric disorders (defined as ICD-10 codes > F40), since these individuals are at particular risk of (major) psychiatric illness onset. Specifically, first-degree relatives are eligible even if diagnosed with a psychiatric disorder categorized within the ICD-10 as F40–49 (anxiety, dissociative, stress-related, somatoform, and other non-psychotic mental disorders), F50–59 (behavioral syndromes associated with physiological disturbances and physical factors), and F60–69 (disorders of adult personality and behavior) codes, as long as these disorders only influence their current state to a minor degree (i.e. they are well-treated at the time of inclusion).

Exclusion criteria for both sub-studies are individuals with intellectual disability (defined as an estimated IQ < 70) [50], schizophrenia or schizoaffective disorder, neurological disorder (including dementia), current alcohol or substance abuse (up to 3 months prior to inclusion), or history of head trauma. To ensure safety of EPO treatment throughout the study, candidates are also excluded if they have significant medical conditions (e.g. heart disease, diabetes, renal failure, untreated/insufficiently treated hypertension, malignancies, and/or thromboses), personal or first-degree family history of epilepsy or thromboembolic events, have received electroconvulsive therapy (ECT) three months before participation, are dyslexic, use contraceptive medications, smoke, are pregnant, or are breastfeeding. Regarding fMRI assessments, participants are not eligible if they suffer from claustrophobia or have a pacemaker and/or other metal implants inside their bodies. Participants, who do not meet these fMRI inclusion criteria will not be excluded from the trial per se, but only from the two fMRI assessments.

These criteria are similar to the procedures in our previous studies [39, 40, 58]. To ensure that participants receive a sufficient concentration of EPO, candidates are excluded if they weigh < 45 or > 95 kg or are overweight (BMI > 30). Participants must be able to provide written informed consent to be included in the study. These procedures are in accordance with the ethical standards of the Danish Research Ethics Committee for the Capital Region of Denmark (protocol number H-16043370) and The Danish Data Protection Agency Capital Region of Denmark (protocol number RHP-2017-020). See Additional file 1 (SPIRIT 2013 Checklist) for a trial protocol checklist.

### Setting

Participants will receive intravenous infusions of either recombinant human EPO (Epoetin alpha; Eprex; 40,000 IU/mL) or placebo (1 mL NaCl) diluted with 100 mL saline (0.9% NaCl) administered for 15 min once a week (7 ± 2 days) during a 12-week study period at the Copenhagen Affective Disorder Research Center (CADIC), Psychiatric Centre Copenhagen, Rigshospitalet. The EPO doses are similar to those found to be effective for modulating neural and cognitive function with short-term administration [46] and for enhancing cognition with long-term treatment [39, 40, 42, 59]. Outcome assessments are also carried out at Psychiatric Centre Copenhagen and Neurobiology Research Unit (NRU), Rigshospitalet.

### Study design and procedures

The trial has a randomized, double-blinded, placebo-controlled, parallel group design. The study design comprises four major assessments (baseline, week 3, week 13, and a six-month follow-up after treatment completion) and weekly safety monitoring and study medication infusions during the 12-week treatment period. Participants will be informed about the study and given a participant information sheet. Before undergoing eligibility assessments, participants provide written informed consent, which will be obtained by one of the named authors.

The baseline assessment is divided into two days, 1–3 days apart for practical reasons and to avoid attrition. On the first day of the baseline assessment, participants are mood rated with the HDRS-17 and YMRS to ensure remission and afterwards complete an fMRI scan at Copenhagen University Hospital, Rigshospitalet. On the second baseline day, participants attend Psychiatric Centre Copenhagen for a fasting research blood test (peripheral biomarker measure) followed by an assessment of cognitive functions with a neuropsychological test battery, verbal IQ (assessed with the Danish Adult Reading Test; DART [56]), and filling in questionnaires concerning subjective cognitive complaints, quality of life, level of functioning, and functional capacity, as well as depression and mania symptom severity ratings. Functional capacity will be assessed using a clinician-rated interview and a performance-based task. Mood ratings are performed with the HDRS-17 [51] and the YMRS [52]. After two weeks of treatment (i.e. two doses of EPO or saline), fMRI scan, research blood samples, neuropsychological testing, mood ratings, and questionnaires on subjective cognitive difficulties are repeated. After treatment completion (week 13) and at the six-month follow-up, the neuropsychological tests, questionnaires concerning subjective cognitive complaints, quality of life, and functional capacity (self-reported and performance-based) are repeated. Research blood samples are collected in week 13, but not at the six-month follow-up. Sleep quantity and quality in the past three days is assessed before each of the four major assessment time points and with online self-rating using the Pittsburgh Sleep Quality Inventory (PSQI) [60] assessing sleep quality one week before the baseline, week 3, week 13, and the six-month follow-up assessment. Blood samples are collected at baseline and weeks 3 and 13 for assessment of potential blood-based biomarkers of pro-cognitive effects. An intermediate mood symptom rating for patients (sub-study 2) is performed at week 7 to assess whether they are in (partial) remission throughout the study period. To ensure safety, we conduct weekly monitoring, comprising thorough medical examinations and blood test evaluations, for the duration of the treatment course and in weeks 13 and 15. Pregnancy tests are mandatory for and will be performed on fertile female participants before the first study medication infusions and every second week during the active treatment period. Blood screening and thorough medical examinations are undertaken at baseline, weekly throughout the 12-week treatment period as well as one and three weeks after treatment completion to continuously monitor red blood cell levels and ensure participant safety.

EPO will be kept at 2–8 °C during transport and storage to reduce the risk of damaging the medication and potential related adverse side effects. EPO will be dissolved in 100 mL saline and infusions will be given intravenously over 15 min to reduce the possibility of acute allergic reactions. If blood test analyses reveal significantly increased hematocrit (men: > 50%; women: > 48%) at two consecutive measurements within the same week, bloodletting (450 mL) will be performed on a weekly basis with no cessation of treatment before hematocrit values are normalized. Patients are therefore asked to regularly drink plenty of water to avoid “pseudo”-increases in hematocrit. In cases of significant increase in thrombocytes (> 400 billion/L) or drop in reticulocytes (ERC(B) < 1 × 10^− 3^), two repeated controls will be performed in the following week. If thrombocyte and/or reticulocyte values remain abnormal, participants will be withdrawn from further study participation and monitored with weekly medical examinations and blood samples until values are stabilized. If necessary, these participants will be hospitalized for observation. Lists of any pseudo-anonymized outcome data collected for participants who discontinue or deviate from further study participation will be kept in a locked cabinet.

### Randomization and blinding

The independent Pharma Consulting Group AB (www.pharmaconsultinggroup.com) has conducted block randomization for each sub-study group. Randomization is performed utilizing a 1:1 allocation ratio. Treatment groups will be stratified for gender and age (sub-study 1: < or > 30 years; sub-study 2: < or > 35 years). At the time of enrolment, diagnosis, gender, and date of birth are registered in order to determine the appropriate stratum to which the participant belongs. Study identification numbers will be given consecutively within each stratum. To ensure blinding of outcome-assessors, sealed randomization envelopes are kept in a locked cabinet only accessible to study personnel responsible for preparing the study medication, who are not involved in evaluation of the efficacy parameters or regular interaction with participants (i.e. the study nurse and PhD student). Double-blinding is achieved during infusion through injection of 1 mL colorless recombinant human EPO (Eprex; 40,000 IU; Janssen-Cilag) or saline (NaCl 0.9%) is injected into a standard 100 mL saline (NaCl 0.9%) infusion bag that will be given to the blinded research nurse or physician administering the study medication within 1 h before drug administration. The weekly safety monitoring of blood tests and side effects will be completed by medical doctors not involved in outcome assessments. To maintain blinding, participants are instructed to not talk about any symptoms or potential physical side effects associated with high-dose EPO treatment (e.g. increased hematocrit or bloodletting) with the study personnel who administer infusions or are involved in outcome assessments. At the last follow-up assessment (after six months), participants and blinded study personnel are asked to guess whether they believe they received EPO or saline treatment during their study participation. This is done to evaluate allocation concealment. Unblinding is permissible for safety reasons in cases of side-effects or serious adverse events likely or directly related to the study medication. The procedure for revealing a participant’s allocated intervention during the trial involves opening his or her randomization envelope. In these cases, it is LSS, MV, or LVK’s sovereign decision, whether the randomization code should be broken. The study is monitored by the Good Clinical Practice Unit (GCP) at the Copenhagen University Hospital (www.gcp-enhed.dk/kbh).

### Outcome assessments

**Fig. 1:**
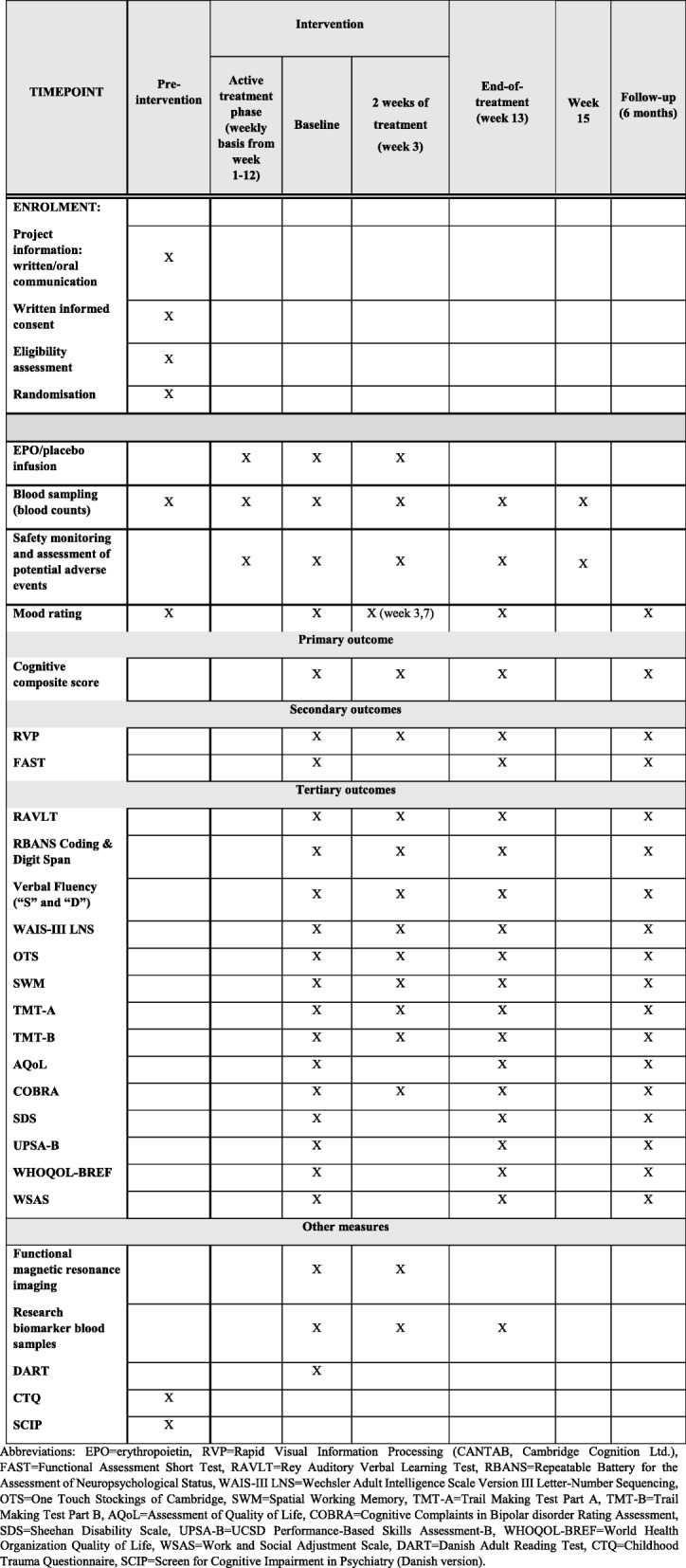
Schedule of enrolment, interventions, and assessments. EPO erythropoietin, RVP Rapid Visual Information Processing (CANTAB, Cambridge Cognition Ltd.), FAST Functional Assessment Short Test, RAVLT Rey Auditory Verbal Learning Test, RBANS Repeatable Battery for the Assessment of Neuropsychological Status, WAIS-III LNS Wechsler Adult Intelligence Scale Version III Letter-Number Sequencing, OTS One Touch Stockings of Cambridge, SWM Spatial Working Memory, TMT-A Trail Making Test Part A, TMT-B Trail Making Test Part B, AQoL Assessment of Quality of Life, COBRA Cognitive Complaints in Bipolar disorder Rating Assessment, SDS Sheehan Disability Scale, UPSA-B UCSD Performance-Based Skills Assessment-B, WHOQOL-BREF World Health Organization Quality of Life, WSAS Work and Social Adjustment Scale, DART Danish Adult Reading Test, CTQ Childhood Trauma Questionnaire, SCIP Screen for Cognitive Impairment in Psychiatry (Danish version)

For an overview of outcome assessment frequency and timing, see Fig. 1. The outcome measures listed below are consistent with the latest recommendations from the ISBD Task Force [50] suggesting the inclusion of a cognitive composite score as the primary outcome measure, a single intervention-specific cognitive measure as the secondary outcome, and multiple individual cognitive measures as tertiary (i.e. exploratory) outcome measures.

#### Primary outcome measures

The primary outcome measure is a cognitive composite score, consisting of neuropsychological tests covering attention, memory, and executive functions. We have previously demonstrated an improvement on this “speed of complex cognitive processing” composite measure in patients with BD after eight weeks of EPO treatment [39]. In the present trial, the specific tests included in the primary composite outcome measure are the Rey Auditory Verbal Learning Test (RAVLT) [61, 62], The Repeatable Battery for the Assessment of Neuropsychological Status (RBANS) Coding [63], Verbal Fluency with the letter “D” [64], Wechsler Adult Intelligence Scale (WAIS)-III Letter-Number Sequencing [65], Trail Making Test Part B (TMT-B) [66], and Rapid Visual Information Processing (RVP) from the Cambridge Neuropsychological Test Automated Battery (CANTAB, Cambridge Cognition Ltd.). To derive the cognitive composite score, we will z-transform and sum performance scores from RAVLT total recall, TMT-B, WAIS-III Letter-Number Sequencing, RBANS Coding, Verbal Fluency (letter “D”), and RVP speed for correct responses using the mean and SD from a healthy control group.

#### Secondary outcome measure

The secondary cognitive outcome measure consists of the RVP (CANTAB), which revealed particularly strong effects of EPO in our previous eight-week study [39], and a functional capacity outcome measure assessed with the clinician-rated interview Functional Assessment Short Test (FAST) [67].

#### Tertiary outcome measures

The tertiary cognitive outcome measure comprises the RAVLT, RBANS Coding and Digit Span, Verbal Fluency with the letters “S” and “D” [64], WAIS-III Letter-Number Sequencing, the One Touch Stockings of Cambridge (OTS; CANTAB), the Spatial Working Memory (SWM; CANTAB), as well as the TMT-B and Trail Making Test Part A (TMT-A) [66]. The tertiary level of psychosocial functioning outcome are the following questionnaires and performance-based task: Assessment of Quality of Life (AQoL) [68], the Cognitive Complaints in Bipolar Disorder Rating Assessment (COBRA) [69], Sheehan Disability Scale (SDS) [70], the UCSD Performance-Based Skills Assessment-B (UPSA-B) [71, 72], the World Health Organization Quality of Life (WHOQOL-BREF) [73], and the Work and Social Adjustment Scale (WSAS) [74]. History of early life stress will be assessed with the Childhood Abuse and Trauma Scale [75] at the time of inclusion.

To minimize learning effects on neuropsychological test performance at the follow-up assessments, alternate versions of the RAVLT (original list AB, GeAB, and Cr-AB) and RBANS Coding and Digit Span (version A and B) [61–63] are used. These versions will be administered in counter-balanced order within each stratum.

### Neural biomarkers of potential pro-cognitive effects

To assess whether an early change in neural activity in the dlPFC and mPFC is predictive of pro-cognitive efficacy, participants will complete MRI scan at baseline and following two weeks of treatment. The MRI scan duration is approximately 1.5 h and includes a structural scan, three functional tasks, including (1) a Strategic Picture Encoding Task, (2) a Verbal task, and (3) a Spatial N-back working memory task from our previous study [45], as well as a checkerboard pattern sequence and a resting state sequence. The fMRI protocol has been constructed to evaluate the sensitivity and specificity of potential neural activity change in the dlPFC and mPFC after two weeks of active treatment and whether this can predict cognitive improvement after 12 weeks of treatment.

### Exploratory measures of potential pro-cognitive effects

To further increase insight into the underlying neurobiological mechanisms involved in the potential beneficial cognitive effects of EPO, blood samples from baseline, week 3, and after treatment will be analyzed for the exploratory purpose of investigating whether baseline levels and/or changes of peripheral biomarkers, including inflammatory markers, brain-derived neurotrophic factor (BDNF), and metabolic parameters (fat and glucose markers), are correlated with cognitive improvement [76–83]. Baseline blood samples will also be used for assessment of potential influence of Catechol-O-methyltransferase (COMT: Val158Met), BDNF: Val66Met, and EPO and EPO-R genotypes on the treatment-related change in cognition and neural activity.

We will perform post-hoc exploratory analyses investigating which demographic, clinical, cognitive, and neural variables at baseline predict treatment efficacy on the primary cognitive outcome measure, since there is a paucity of research into which baseline factors are associated with cognitive improvements [84].

### Biochemistry

Research blood samples will be transferred to the Neuropsychiatric Laboratory, Rigshospitalet, and stored at − 80 °C until use. Measurements will be performed at Neuropsychiatric Laboratory, Department O, and at Department of Clinical Pharmacology, Rigshospitalet.

### Statistical analyses

The threshold for statistical significance is considered *p* < 0.05 (two-tailed). For significant results regarding the primary, secondary, and tertiary measures, relevant effect sizes will be reported in addition to the *p* values. All statistical analyses are performed using the Statistical Package for Social Sciences (SPSS, version 23, IBM Corporation, Armonk, NY, USA).

#### Primary, secondary, and tertiary outcome measure analyses

Behavioral data from neuropsychological test score performance, subjective cognitive impairments, quality of life, level of functioning, psychosocial functioning, and mood symptoms (i.e. data from the primary, secondary, and tertiary outcomes) will be analyzed using mixed models design and intention-to-treat (ITT) analyses in cases of missing data. Data will be analyzed for every participant with any assessment. No interim analyses will be performed.

#### Functional MRI analyses

Functional MRI data are pre-processed and analyzed with FMRIB Expert Analysis Tool (FEAT) and the “randomize” algorithm implemented in FSL (FMRIB Software Library; www.fmrib.ox.ac.uk/fsl). We will assess whether early differences between EPO and placebo groups in task-related neural activity during each of the three fMRI paradigms after two weeks of treatment (adjusted for baseline activity) predict subsequent treatment efficacy on cognition at treatment completion. Neuropsychological test performance and fMRI data from 40 cognitively intact healthy controls without personal or first-degree relative history of mental illness from the BIO study [85] is used as external normative data.

Region of interest (ROI) analyses of fMRI data from the N-back working memory tasks will be carried out to investigate the effects of EPO on neural activity in dlPFC. The difference in neural activity between the EPO and saline groups in week 3 will be investigated by extracting and analyzing mean percent signal change in dlPFC adjusted for potential differences in baseline activity using univariate analysis of covariance (ANCOVA) and with whole-brain analyses (FEAT) in week 3 (with adjustment for potential activity differences at baseline). We will also investigate the hypothesized early dorsal PFC activity change in response to EPO versus saline with volume of interest (VOI) for the dorsal PFC. Volume of interest analyses of dorsal PFC and the hippocampus are conducted to assess fMRI data from the Strategic Picture Encoding Task. Finally, exploratory whole-brain analyses are conducted to assess treatment-related activity change in other brain regions. Differences in neural activity between groups will be correlated with potential change in the primary cognitive composite score at week 3 and after treatment completion. If this correlation is significant, multiple regression analyses will be performed with adjustment for mood symptoms, age, and gender to assess the potential predictive value of early neural activity change for potential pro-cognitive efficacy after 12 weeks of EPO treatment.

#### Peripheral blood-based biomarker analyses

Post-hoc analyses will be conducted on research blood sample data collected at baseline, week 3, and week 13 for the exploratory purposes to assess whether potential efficacy on cognition in response to EPO versus saline is accompanied by and related to changes in blood-based biomarkers of inflammation and metabolism.

#### Sample size and power calculation

Sample size and statistical power has been calculated by PharmaConsulting Group AB with Statistical Analysis Software (SAS), based on our previous findings assessing cognitive effects of weekly infused EPO [48]. The difference in cognitive change between the EPO and the saline-treated groups from baseline to after treatment was 0.5 SD [48]. In this trial, we estimate a clinically relevant differential change in the primary cognitive composite score between the EPO and placebo groups from baseline to week 13 (treatment completion) to be at least 0.4 SD (corresponding to a moderate effect size) with a SD of the mean change of 0.5 between these groups. This is consistent with the recommendations listed by the ISBD Cognition Task Force [50]. Specifically, the task force indicated that a differential change between groups of 0.2–0.4 SD on a global composite score reflects a potentially clinically relevant change, since this may translate into moderate–large functional improvement in patients with mood disorders [50]. In our eight-week EPO trial, the difference regarding change in the cognitive composite score from baseline to treatment completion was 0.5 SD between the EPO and the saline groups [48]. Based on the ISBD task force recommendations and our earlier findings regarding effects of longer-term EPO treatment, we estimate that a sample size of *N* = 104 (i.e. *n* = 52 participants within each sub-study with *n* = 26 in each treatment group) will reach a ≥ 80% power for detecting a similar clinically relevant differential change of 0.4 SD in the primary cognitive composite outcome measure with a SD of this change of 0.5 between the treatment groups at an alpha level of 5% (two-sided test). Based on our assumption of a 10% drop-out rate from baseline to treatment completion, we plan to recruit up to *n* = 58 for each sub-study to achieve complete datasets for *n* = 52 participants per sub-study (first-degree relatives and patients, respectively).

#### Data management and monitoring

All personal information will be obtained at the eligibility assessment or from patient records, if patients are unable to provide the necessary pieces of information. Written informed consent forms will be kept in a locked filing cabinet, while a password-protected list that matches participant ID numbers with personal information will be stored separate from pseudo-anonymized data. The list matching participants’ personal information with their ID number will be deleted and consent forms maculated 10 years after study completion. At this point, all data will be completely anonymized. All trial authors will have access to the final trial dataset. Pseudo-anonymized research data will be registered in the Research Electronic Data Capture (REDCap) database, which fulfills the Danish data law to keep research participants’ records and meets GCP requirements for data management. Study personnel involved in outcome assessments and evaluation of these outcomes are blinded to study medication until the data analyses are completed. Consequently, blood sample results and lists of potential adverse effects are registered in The Healthcare Platform (Sundhedsplatformen) to which only medical doctors responsible for participant safety and the person involved in blinding of the study medication have access. REDCap has a logging module, which enables tracking of the data entered. Data quality is ensured by verification of data entered by outcome assessors and score range restrictions on values from neuropsychological test and questionnaire results. All neuropsychological data for the primary outcome measure will be double-checked by JZP.

#### Participant retention

All participants are offered feedback on the changes of their neuropsychological performance across assessment time points once they have completed the six-month follow-up assessment. Furthermore, employed participants will be given a compensation of 100 DKK per hour for 10 h of neuropsychological and fMRI assessments. Travel expenses with public or private transportation are reimbursed for all participants. Finally, patients will benefit from the extra care they receive from study nurses, psychologists, and medical doctors during their participation.

## Discussion

### Summary

Cognitive dysfunction is a core feature of BD and UD, which reduces socio-occupational functioning. The lack of clinically available treatments with pro-cognitive efficacy is partially related to major methodological challenges in this relatively new field, including the absence of a sensitive brain-based biomarker model to select among candidate treatments. Preclinical and clinical studies point to EPO as one of the most promising candidate cognitive enhancement treatments, making it a suitable potential treatment for assessment of neural activity change associated with improvement of cognition. The aim of the present trial is therefore twofold: (1) to clarify whether 12 weekly EPO versus placebo (saline) treatment has pro-cognitive effects in cognitively impaired remitted patients with BD or recurrent UD and first-degree relatives without mood or psychotic disorders to patients with BD, recurrent UD, or schizophrenia; and (2) to identify patterns of early treatment-related neural activity change that may be predictive of subsequent cognitive improvement.

### Strengths

The present EPO trial includes, for the first time, first-degree relatives with cognitive impairment (sub-study 1), which will clarify whether EPO has the potential to improve cognitive and functional outcome in genetically predisposed individuals, who are at increased risk of stress and illness onset [86]. In general, the present trial (both sub-studies) has several advantages over our previous EPO cognition trial in BD and TRD [39, 40]. Notably, the previous trial did not involve pre-screening for objectively verified cognitive dysfunction, which may have introduced type II errors since 30–50% of bipolar patients and 60–80% of depressed patients are relatively cognitively intact compared with neuropsychological test norms for age-matched individuals, despite frequent subjective cognitive complaints [1, 2, 87]. In the present trial, we will therefore only include participants who present objective cognitive dysfunction on a brief cognition screening tool (SCIP) in keeping with the recent methodological recommendations by the ISBD Targeting Cognition Task Force [50]. Due to the cognitive heterogeneity characterizing remitted patients with BD and UD [87], the ISBD Cognition Task Force recommends a broad cognitive composite score spanning attention, memory, and executive function as primary outcome in cognition trials in mood disorders [50]. In keeping with this, we have chosen the “speed of complex cognitive processing” composite as the primary outcome. While the longest follow-up in our previous EPO trial was only six weeks after treatment completion, we here include a six-month follow-up assessment to assess whether any treatment-related cognitive improvements persist long term. Further, the inclusion of self-reported, observer-rated, and performance-based measures of functional capacity will provide insight into whether potential cognitive benefits of EPO translate into improved daily functioning, which is the ultimate goal for our patients. Finally, it is likely that the present expansion of the treatment period from 8 to 12 weeks will result in more robust and longer-lasting effects of EPO treatment. Beyond EPO, the use of fMRI to assess early treatment-related change in neuronal activity within regions of the “cognitive control network” and the correlation between such change and subsequent cognitive improvement will provide insight into the neurobiological targets of potential cognitive improvement and thus aid future treatment development strategies [50, 88].

### Limitations

The disadvantage of only enrolling participants with objectively verified cognitive dysfunction is that this limits recruitment, since many patients with mood disorders are relatively cognitively intact in comparison with norms [1–3, 87]. For these reasons, we selected a cut-off on the brief cognition screening tool (SCIP) to ensure the presence of minimum subtle cognitive impairments in this trial. Due to the extensive somatic co-morbidity exclusion criteria, the study sample may not represent the full range of BD and recurrent UD, which limits generalizability of findings. However, this is necessary to ensure participant safety, which is of principal importance in the trial. Notwithstanding, this highlights the major limitation of this treatment, since a large proportion of patients have somatic co-morbidities and EPO may therefore only become relevant for treatment of cognitive impairments in a subgroup of patients (if the study finds positive effects). This highlights a need for development of other kinds of treatments to improve cognition such as action-based cognitive remediation (ABCR) [89]. The thorough assessments before and during study participation may contribute to a selection of participants who are more positive towards clinical research and therefore also more willing to cooperate. Indeed, this may partially explain the very high compliance in our previous EPO trial, in which there was only one drop-out [39]. Finally, patients with BD and recurrent UD will be on medication for ethical reasons and to aid generalizability of the results [50]. However, this concomitant pharmacological treatment may confound neuropsychological test and fMRI task data due to possible effects of medications on cognition and BOLD response [90]. We therefore seek to minimize confounding effects by avoiding changes in patients’ concomitant medication during the study period, if possible, and by carefully recording their medication, so potential interaction effects with EPO can be assessed in post-hoc analyses. Further, we anticipate an equal distribution of medications between treatment groups as in our previous EPO studies [39, 40], so any differences in cognition and neural change between groups will be due to EPO or saline.

### Study feasibility

We have previously conducted a double-blinded randomized trial of eight weeks of EPO treatment in 84 patients with mood disorders at the Psychiatric Centre Copenhagen, Rigshospitalet. Based on this trial as well as our collaboration on recruitment with other psychiatric centers and consultant psychiatrists in the Capital Region of Denmark, we consider recruitment of 52–58 patients and 52–58 first-degree relatives over 30 months to be feasible.

### Safety procedures and monitoring of EPO infusions

EPO is a common treatment option for anemia patients and has a good safety profile when carefully monitored. However, hematopoietic effects of repeated EPO administration are associated with risk of hypertension and blood clotting [91]. Indeed, EPO has been associated with increased mortality in severely ill stroke patients with previous thromboembolic disease, including patients given thrombolytic treatment [92, 93]. To ensure participant safety in this trial, we therefore implement extensive exclusion criteria to exclude candidates at increased risk of thromboembolic events. Extremely rare but serious side effects associated with long-term EPO administration in patients with chronic severe somatic diseases are thromboses at the site of dialysis in patients with kidney failure, seizure, and potential tumor growth [94]. The rare condition, pure red cell aplasia (PRCA), has been detected with subcutaneous infusions and poor packaging of the EPO medication. However, its incidence rate has fallen to 0.3/100,000 patient years [95–97]. Reticulocyte counts constitute the first indicator of PRCA and are therefore thoroughly monitored. We observed no serious adverse events in eight weeks of weekly EPO infusions in the proposed dose and administration form in our previous studies of 84 patients with TRD or BD [39, 40]. EPO-related hematocrit levels increased to an extent that necessitated blood-letting in five (14%) of the 35 EPO-treated patients in weeks 3–8 (with one in week 3). This corresponds to observations by Ehrenreich et al. [41, 42]. We discontinued EPO treatment in six patients (17%) after 5–7 weeks due to thrombocyte level increase (these participants completed all assessments). Participant safety monitoring therefore involves thorough medical examinations, systolic and diastolic blood pressure measurement, blood sampling, electrocardiography (ECG), and additional safety parameters at baseline, weekly during the study, and at three weeks after EPO/placebo treatment completion. Due to careful adherence to the exclusion criteria and weekly safety monitoring, we evaluate the risk of such side effects and adverse events of EPO treatment to be extremely low in this trial. Further, participants will be informed of all potential adverse effects before randomization and are instructed that iron supplements (which increase hematocrit levels) are prohibited during the active treatment period. Although the risk of potential thrombosis or suspected PRCA is low, included participants are given a pocketsize plastic card with instructions about what to do and contact details to medical doctors at the local emergency department in case these symptoms appear. Participants stay at the clinic for observation for at least 30 min after each infusion for a research nurse to monitor potential acute side effects of EPO (e.g. rash at the infusion site or headache).

### Ethical considerations

We evaluate the risks and disadvantages of participating in the study to be minimal based on previous EPO trials [39, 40] and the described precautions, exclusion criteria, and carefully established treatment plan in case of side effects.

Although patients are not required to withdraw from their usual antidepressant or mood stabilizing medication, they are requested not to change their doses or treatment during the study course. This may give rise to ethical considerations for the included remitted patients, albeit we show consideration for their safety, rights, and wellbeing by excluding those who are required to change the type or dose of their mood stabilizing medical treatment by their psychiatrist. In case of significant symptom worsening, patients’ clinical needs, integrity, and autonomy come before the scientific interests of the study. Because of the lack of effective treatments targeting cognitive dysfunction in mood disorders [22, 98], the use of a placebo group is necessary for investigating potential beneficial cognitive effects of EPO. We are unable to offer active EPO treatment to these participants after trial completion given the only preliminary evidence for efficacy of EPO on cognition. This is likely to be disappointing for the 50% of participants randomized to saline. The duration of neuropsychological test assessments and mood ratings may lead to attrition in some participants. However, we keep the duration of neuropsychological assessments and fMRI scans to a minimum to avoid attrition and reduce the risk of drop-out. Albeit the procedure is safe and non-invasive, some participants may experience it as claustrophobic and anxiety provoking. For these reasons, we exclude people, who suffer from claustrophobia from fMRI assessments. Blood sampling may be associated with slight discomfort but is routine hospital care. Participants will benefit from the extra care and close contact with medical doctors, psychologists, and a research nurse during study participations, which has demonstrated beneficial effects [99]. Furthermore, the participation in the trial is considered beneficial for first-degree relatives to patients with BD, recurrent UD, or schizophrenia, since enrolment requires objectively verified cognitive dysfunction, which is known to increase psychiatric illness onset risk in these high-risk individuals [20]. All participants are reimbursed for their time and the transport expenses associated with taking part in the study.

### Perspectives

If the findings reveal pro-cognitive efficacy of EPO and that this is associated with early prefrontal activity change, this would (1) point to EPO as a candidate compound targeting cognitive dysfunction in somatically healthy patients with mood disorders and (2) highlight prefrontal target engagement as a promising biomarker model for pro-cognitive efficacy. From a methodological perspective, such findings will be an important step in future development of cognitive enhancement treatments in neuropsychiatric disorders and preventive strategies in at-risk populations, which could have significant individual and societal implications.

## Trial status and dissemination

Participant enrolment was initiated in September 2017 and is expected to continue until autumn 2020. Follow-up data from the last recruitments will be collected during the winter of 2021. Trial results will be disseminated in peer-reviewed scientific journals and presented at scientific conferences and meetings. Author eligibility is assessed with the Vancouver Convention.

## Additional file

Additional file 1: SPIRIT 2013 Checklist: Recommended items to address in a clinical trial protocol and related documents*. (DOC 125 kb)

## Abbreviations

ABCR: Action-based cognitive remediation
ANCOVA: Analysis of covariance
AQoL: Assessment of Quality of Life
BD: Bipolar disorder
BDNF: Brain-derived neurotrophic factor
BOLD: Blood-oxygen-level dependent
CANTAB: Cambridge Neuropsychological Test Automated Battery
COBRA: Cognitive Complaints in Bipolar Disorder Rating Assessment
COMT: Catechol-O-methyltransferase
DART: Danish Adult Reading Test
dlPFC: Dorsolateral prefrontal cortex
dmPFC: Dorsomedial prefrontal cortex
dPFC: Dorsal prefrontal cortex
ECG: Electrocardiography
ECT: Electroconvulsive therapy
EPO: Erythropoietin
FAST: Functional Assessment Short Test
fMRI: Functional magnetic resonance imaging
HDRS-17: Hamilton Depression Rating Scale (17-items version)
ISBD: International Society for Bipolar Disorders
ITT: Intention to treat
mPFC: Medial prefrontal cortex
OTS: One Touch Stocking of Cambridge
PFC: Prefrontal cortex
PRCA: Pure red cell aplasia
PSQI: Pittsburgh Sleep Quality Inventory
RAVLT: Rey Auditory Verbal Learning Test
RBANS: Repeatable Battery for the Assessment of Neuropsychological Status
RCT: Randomized controlled trial
ROI: Region of interest
RVP: Rapid Visual Processing
SCAN: Schedules for Clinical Assessment in Neuropsychiatry
SCIP: Screen for Cognitive Impairment in Psychiatry
SPSS: Statistical Package for Social Sciences
SWM: Short Working Memory
TMT: Trail Making Test
TRD: Treatment-resistant depression
UD: Unipolar disorder
UPSA-B: UCSD Performance-Based Skills Assessment-B
VOI: Volume of interest
WAIS: Wechsler Adult Intelligence Scale
WHOQOL: World Health Organization Quality of Life
WSAS: Work and Social Adjustment Scale
YMRS: Young Mania Rating Scale
